# Quantum receiver enhanced by adaptive learning

**DOI:** 10.1038/s41377-022-01039-5

**Published:** 2022-12-08

**Authors:** Chaohan Cui, William Horrocks, Shuhong Hao, Saikat Guha, Nasser Peyghambarian, Quntao Zhuang, Zheshen Zhang

**Affiliations:** 1 James C. Wyant College of Optical Sciences, University of Arizona, Tucson, AZ 85721 USA; 2 Department of Materials Science and Engineering, University of Arizona, Tucson, AZ 85721 USA; 3 Department of Electrical and Computer Engineering, University of Arizona, Tucson, AZ 85721 USA; 4 Department of Electrical and Computer Engineering, University of Southern California, Los Angeles, CA 90089 USA; 5 Department of Electrical Engineering and Computer Science, University of Michigan, Ann Arbor, MI 48109 USA

**Keywords:** Single photons and quantum effects, Fibre optics and optical communications

## Abstract

Quantum receivers aim to effectively navigate the vast quantum-state space to endow quantum information processing capabilities unmatched by classical receivers. To date, only a handful of quantum receivers have been constructed to tackle the problem of discriminating coherent states. Quantum receivers designed by analytical approaches, however, are incapable of effectively adapting to diverse environmental conditions, resulting in their quickly diminishing performance as the operational complexities increase. Here, we present a general architecture, dubbed the quantum receiver enhanced by adaptive learning, to adapt quantum receiver structures to diverse operational conditions. The adaptively learned quantum receiver is experimentally implemented in a hardware platform with record-high efficiency. Combining the architecture and the experimental advances, the error rate is reduced up to 40% over the standard quantum limit in two coherent-state encoding schemes.

## Abstract (web summary)

The proposed adaptive-learning framework empowers automatic design of quantum receivers capable of combating noise in distinguishing large-alphabet nonorthogonal quantum states. Experiments show up to 40% improvement over standard approaches.

## Graphical abstract

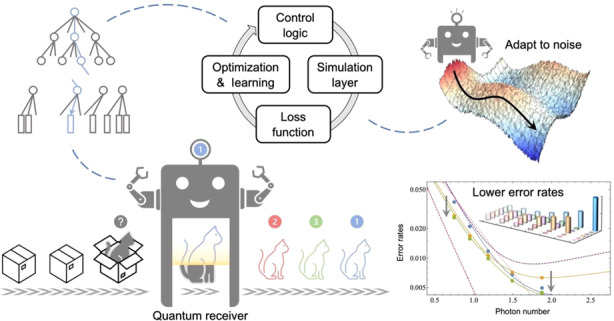

## Introduction

Quantum information science (QIS) endows communication^1–3^, sensing^4,5^, and computing^6,7^ capabilities unrivaled by their classical counterparts. QIS has also sharpened our understanding of the fundamental limits of information acquisition, transfer, and processing due to the indistinguishability of nonorthogonal quantum states, which in turn place bounds on the rate of optical communications^8,9^ and the precision of sensing^10^. Unfortunately, in many scenarios, an appreciable gap separates the performance achievable by routine measurement apparatus and what is allowed by quantum mechanics^11,12^. A central theme of QIS is thus the quest for protocols that approach the ultimate performance limits.

Quantum receivers are unconventional measurement apparatuses designed to bridge the gap and enable a performance boost in a wide range of information processing tasks modeled as quantum-state discrimination^13^ or parameter estimation^14^. Pioneering quantum-receiver works unveiled that adaptive structures based on quantum circuits configured by feed-forward controls^15,16^ can vastly reduce the error probability in discriminating nonorthogonal quantum states. A landmark development was the Dolinar receiver capable of discriminating two weak coherent states at the fundamental Helstrom bound^17^. More recent studies have laid out quantum-receiver structures to benefit a variety of near-term tasks encompassing quantum state tomography^18–21^, target detection^22,23^, communication^24–31^, and computing^32,33^. Despite these encouraging advances, the design of quantum receivers for general QIP problems remains a formidable challenge, primarily due to the large Hilbert space that quantum states reside in. Indeed, only a few are known to achieve the ultimate performance limit even excluding imperfections. In practical situations, noise and disturbance in a dynamic environment would further bring substantial complexities to the quantum-receiver design^34^, rendering the traditional design method based on analytic modeling clumsy and impotent.

The rapid development of data science has given rise to efficient tools for addressing complex data-processing problems in a large parameter space, shifting the landscape of data mining^35^, computer vision^36^, automated control^37,38^, and decision-making^39^. State-of-the-art data-science methods now help combat the unprecedented challenge of designing quantum protocols and platforms, such as engineering of quantum states^40,41^, operators^42^, or a combination of both^43,44^.

Here, we harness reinforcement learning^45^ to design quantum receivers, formulating the quantum receiver enhanced by adaptive learning (QREAL) architecture capable of tackling a series of quantum-state discrimination and data-classification problems. The QREAL architecture enjoys a substantially reduced complexity compared with other proposals based on model-free reinforcement learning^46,47^, allowing it to be embodied in a photonic platform compatible with telecommunication. We then verify QREAL’s capability of tackling different quantum-state discrimination problems with advantages over the standard quantum limit (SQL). By virtue of its capability of adapting to diverse operational conditions, QREAL’s advantage over prior quantum receivers is further magnified in the presence of practical imperfections. These features of QREAL render it readily available to enhance long-haul communications, imaging, and sensing systems.

### The QREAL Architecture

**Fig. 1:**
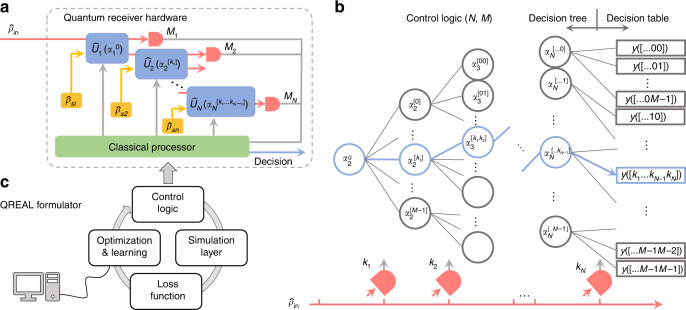
Overview on the QREAL architecture. **a** The hardware of a general quantum receiver composed of rounds of processing. The *j*th processing round entails a variational quantum circuit represented by the unitary operation $\hat U_j$ configured by the parameter set *α*_*j*_, ancillary states $\hat \rho _{s_j}$, and measurement apparatus ${{{\mathcal{M}}}}_j$. **b** The control logic comprised of a decision tree and a decision table. Blue lines show one possible path toward the output. Quantum-receiver output *y* is determined by the sequence of measurement outcomes, tied to a unique path in the decision tree. **c** The QREAL formulator consisting of four modules (clockwise) as one iteration in the learning process. The control logic optimized by the QREAL formulator is compiled and built into the classical processor

The general goal of our quantum receiver is to perform a hypothesis-testing task between a set of quantum states, each tagged by a classical entry *y*. A general quantum-receiver structure depicted in Fig. 1a comprises *N* rounds of processing and measurements. Following the control logic, the classical processor manages the quantum circuit and provides a decision based on the measurement history. In the *j*th round, the quantum state from previous steps is modified by a history-configured unitary operation $\hat U_j(\alpha _j^{\left[ {k_1k_2 \ldots k_{j - 1}} \right]})$ with an ancillary state $\hat \rho _{s_j}$, then mapped into a result *k*_*j*_, which leads to the operation in the next round.

Figure 1b illustrates a control logic comprised of an *M*-ary decision tree with depth *N* and a decision table, both employed by a quantum receiver specified as QREAL (*N*,*M*). Each node, linked from its parent node, contains a variable that is optimized by the QREAL formulator (Fig. 1c). The noise awareness of QREAL is accomplished by simulating a batch of decision trees, each generated with stochastically sampled noise through all rounds. In doing so, the QREAL formulator obtains a collection of probability distributions for the measurement history conditioned on each input state, subject to the noise characteristics. Then, the decision table is derived by Bayesian inference, and the decision tree is adjusted to lower the loss function. After the iterations, an optimized control logic for a specific QIP task is loaded into the classical processor (Detailed description in supplementary section I).

In such an iterative quantum processor with feed-forward, noise is detrimental since its impact may accumulate through operations. Noise can carry different characteristics within the time scale of a round. High-frequency noise uniformly degrades the fidelity of unitary operations in each round, which can be simply treated as independent and get modeled easily. When noise varies on a time scale much longer than *N* rounds, it can be regulated by tracking the drift. However, mid-frequency noise affects the system collectively since it varies on a time scale of several rounds. This type of noise obeys stationary statistics across a batch of decision trees. QREAL aims to learn the pattern of mid-frequency noise and accordingly adapt its measurement strategy toward a better overall performance.

### Setting up QREAL for decoding weak coherent light

The nonorthogonality of quantum states precludes them from being perfectly discriminated, forbidding the classical receivers operate near the fundamental limits. To bridge the gap, we leverage the QREAL formulator to construct quantum receivers for the problem of discriminating weak nonorthogonal coherent states. To date, experimentally implemented quantum receivers are predominantly devised by static approaches that are incapable of adapting to diverse operational conditions, including various genres of noise and imperfections, rendering their performance far inferior to that of QREAL as we show below.

The quantum-receiver hardware is a fiber-based platform operating at the c-band of optical communication. The components realize suitable functionalities within the general QREAL architecture. Specifically, practical modules of variational quantum circuits and measurements are assembled to manipulate and detect weak coherent states.

We next elaborate on the QREAL architecture with more details on the experimental setup, the modeling of noise patterns, and the learning process. In the quantum-receiver hardware, the ancillaries $\hat \rho _{sj}$ are vacuum states. The unitary operation $\hat U_j$ at each round is a reconfigurable quadrature displacement operation with a history-dependent complex variable $\alpha _j^{\left[ {k_1k_2 \ldots k_{j - 1}} \right]}$ for the displacement. The measurement at each round is a photon-number resolving (PNR) detection.

In the optical part of the hardware, the displacement operation is implemented by interfering the signal and a local oscillator (LO) on a 99:1 beamsplitter. During each round of decoding, the relative phase and amplitude of the LO determine the equivalent displacement applied to the signal. The displaced optical field is then captured by a time-multiplexed superconducting single-photon detector. A 5 kHz clock switches the system status between decoding and phase-locking while synchronizing other supplementary electronic devices.

The classical processor is an FPGA implementing the control logic with a decision tree. The FPGA stores an updatable look-up table in its memory. After acquiring photon arrival times from the detector, the FPGA searches and determines the displacement for the next round. It then sends voltage signals to change the phase and amplitude of the LO in a proper time window. The FPGA communicates with a desktop computer that runs the QREAL formulator.

In setting up for a QIP task, the QREAL formulator works with the FPGA to estimate on the incoming signal power, dark count rate, and the photon statistics associated with different displacements on each signal. The average interference fringe visibility is obtained at the same time, as a statistical indicator for the fluctuation and noise. In general, the noise model can embody any pattern.

In the current experiment, the preset noise model fits the statistical outcomes by two individual Gaussian distributions on both the phase and amplitude in the displacement operation and one Poissonian distribution for the dark count and ambient light at the detector. The noise is identical for *N* rounds in each decoding frame but constitutes a stationary stochastic process between frames. The noise model is suitable for our setup since the switch between phase-locking and decoding will eventually add phase fluctuations to each cycle. The intractable loss fluctuation caused by the fiber-stretcher and polarization managements also induces cycle-wise amplitude bias to the LO. Due to such noise, the overall fidelity of displacement operation barely exceeds 99.7%.

Subsequently, based on the pre-estimated noise distributions, the formulator generates a batch of noisy decision trees, each obeying the modeled noise patterns. The formulator then starts to optimize displacements, aimed at reducing the average error rate for the batch of decision trees. (More details are in the supplementary section II, III).

## Results

**Fig. 2:**
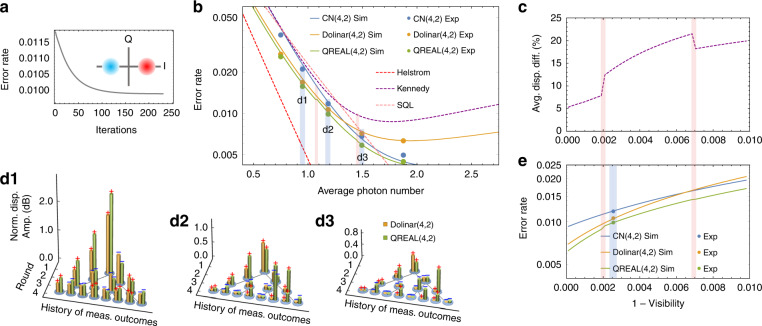
QREAL for BPSK-encoded quantum-state discrimination. **a** Evolution of error rate during learning iterations. Inset: constellation diagram of BPSK encoding. **b** Error-rate comparison for the QREAL and other receivers in the presence of noise. An interference visibility of 99.75% is set in simulations (solid lines) and is measured in experiments (points) for all tested receivers. The Helstrom bound and SQL (homodyne) are noise-free and loss-free. **c** Average difference in displacement amplitudes applied by the QREAL and Dolinar receiver at sub-unity visibilities, showing QREAL’s ability to adapt to imperfections. The corresponding error rates are displayed in **e**. Red belts mark discontinuities in quantum-receiver strategies, a sign of escaping local optimums. **d1–d3**, show the displacement amplitudes (pillars) and phases (signs) of each node in the Dolinar(4,2) and QREAL(4,2) decision trees at the three different input photon numbers (blue belts in **b**). The displacement amplitudes are in a dB scale, normalized to these used in the CN receiver. Mean photon number in producing figure **a**, **b**, and **e** is ~1.2

As a first test, we develop and implement the QREAL robust against noise and other imperfections for the binary phase-shift keying (BPSK) format (Fig. 2a, inset). The discrimination of quantum states encoded in the BPSK format has been extensively investigated, with many quantum receivers proposed^16,17,48^ and implemented^24^ to beat the SQL. The original Dolinar receiver^17^ was the first to approach the ultimate Helstrom bound for BPSK quantum-state discrimination in an ideal scenario, given a mean photon number including loss. However, its advantage over SQL quickly fades away under noise, due to the lack of a mechanism to cope with practical nonidealities when the displacement are preset.

In contrast, QREAL(4,2) for BPSK can mitigate the adverse effect caused by noise and other imperfections by *adaptively* learning their patterns in ~150 iterations (~15 s) (Fig. 2a). The disparity between the learned displacement parameters and those used in the Dolinar(4,2) receiver is shown in Fig. 2c, d1–d3. To verify QREAL(4,2)’s robustness against noise, we test QREAL(4,2), the conditional-nulling^25^ receiver (CN(4,2)), and Dolinar(4,2) subject to an identical noise pattern. The simulation results in Fig. 2b, e show that, while noise quickly degrades the performance of Dolinar(4,2), QREAL(4,2) maintains its advantage over SQL within a broad range of power levels of the input quantum states. In the large photon-number or high-noise regime, QREAL picks a strategy closer to that of the CN receiver, whereas in the small photon-number or low-noise regime, the QREAL learns a strategy closer to that of the Dolinar receiver. Overall, QREAL outperforms the other two quantum receivers across the entire parameter space, shown by the experimental error rates displayed in colored dots, with error bars smaller than the size of the points. With no additional components between the displacement operation and the detector, the experimental setup achieves two parameters to beat BPSK’s loss-less SQL^49,50^, i.e., an overall efficiency of 85% while maintaining the visibility at 99.75%. Specifically, we achieve a raw bit error rate of <2.5% at a mean photon number of ~0.75, corresponding to an information rate as high as 1.1 bits per received photon. QREAL enjoys an error rate 39% below the SQL at a mean photon number of 0.95 and keeps an advantage over the SQL for mean photon numbers below 1.6. After adapting to the noise, QREAL reduces up to 14% error rate compared with the best of the CN and Dolinar receivers.

**Fig. 3:**
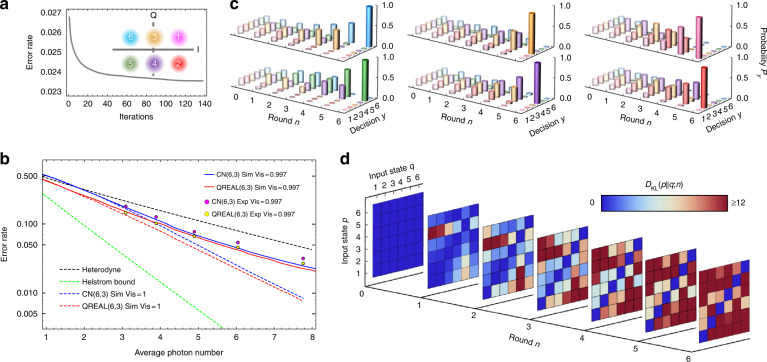
QREAL for QAM-encoded quantum-state discrimination. **a** Evolution of error rate during learning iterations. Inset: constellation of QAM-6 encoding. **b** Comparison of the error rates for QREAL and other receivers in the presence of noise. An interference visibility of 99.7% is set in simulations (solid lines) and is measured in experiments (points) for all tested receivers. Dashed lines are simulated error-rate lower bounds without noise. **c** Posterior probabilities for input quantum states in different rounds. The uniform prior probabilities are plotted in Round 0. The position arrangement and color of states follows the inset in **a**. **d** Evolution of the relative entropy $D_{{{{\mathrm{KL}}}}}(p||q;n)$ (Kullback-Leibler divergence) for the pair-wise measurement statistics conditioned on the input quantum states $|\beta _p\rangle$ and $|\beta _q\rangle$. Mean photon number used in figure **a**, **c**, and **d** is ~7.8

We next develop the QREAL for the quadrature amplitude modulation (QAM) encoding (Fig. 3a, inset) to demonstrate its capability of handling complex tasks in the presence of noise. QAM profits the spectral efficiency by leveraging a larger codeword space but challenges the design of sub-SQL quantum receivers due to its larger parameter space. The QREAL formulator takes ~120 iterations (~10 minutes) to converge the design at a minimal error rate (Fig. 3a). The constructed QREAL surpasses the SQL and beats the error rate of the CN receiver. The learned QREAL(6,3) for QAM-6 decoding is tested back-to-back with CN(6,3). Both the simulation and the experimental results illustrate QREAL’s noise mitigation capability, which underpins QREAL’s performance gain in the low photon-number regime where noise constitutes a significant portion of error events. In practice, QREAL enables up to 43% (average 32%) error-rate reduction over the SQL (loss-free heterodyne) and up to 19% (average 17%) improvement over the CN receiver, as depicted in Fig. 3b.

To illustrate the internal workflow of QREAL, Fig. 3c plots the evolution of the posterior probability distributions through the processing rounds for each codeword, starting from a uniform prior probability distribution. More information about the incoming quantum state is acquired as the QREAL executes through the consecutive rounds so that the correct codeword becomes prominent the posterior probability distribution while the incorrect guesses are suppressed. To further understand how the QREAL discriminates between different codewords, we quantitatively compare the distance between the measurement statistics resulted from the 6 different coherent states using the relative entropy $D_{{{{\mathrm{KL}}}}}(p||q;n)$, as shown in Fig. 3d. By the end of the first two rounds, the measurement statistics for a subset of the codeword states, e.g., $|\beta _1\rangle$ and $|\beta _5\rangle$, have been adequately disparate so that they are distinguished with confidence. The rest of the codeword states, however, remain unsure due to their close measurement statistics. As the processing proceeds into subsequent rounds, more measurement outcomes lead to distinct measurement statistics for different codeword states, allowing QREAL to pick the correct codeword.

We have developed QREAL to address two quantum-state discrimination problems. First, QREAL for BPSK is shown to outperform both the Dolinar and the CN receivers. Notably, QREALs are proven robust against imperfections so that QREAL’s performance advantage over conventional quantum and classical receivers sustains over a large noise region. Such an improvement makes QREAL outperform other quantum receivers in nonideal environments. We also constructed QREAL for QAM to verify its capability of undertaking a complex QIP task that require optimization in a large parameter space with more than 1000 variables. The QREAL architecture is envisioned to endow new functionalities in the noisy intermediate-scale quantum era by harnessing the hybrid quantum-classical information processing architecture.

## Discussion

The quantum-receiver hardware can be augmented with new types of variational quantum circuits and measurements to embrace a wider scope of QIP problems. For example, continuous-variables measurements such as the homodyne detector offers an additional degree of freedom to access a richer set of information^20^. With a versatile topology, the QREAL formulator can be generalized to most near-term measurement-based quantum applications, including quantum state generation^32^, tomography^19^, variational quantum eigensolvers^51^, and quantum-enhanced sensing^23^, after proper discretization.

Moving toward practical operational environments, the QREAL formulator can fit the in-situ learning iterations as tests on the hardware also provide the sampled probability distribution. In doing so, the QREAL formulator will be more efficiently in dealing with the exponentially large quantum-state space and, in the meantime, adapt to slowly-varying noise. This feature enables long-time unsupervised stability, which is crucial for real-world applications. In addition, the QREAL formulator supports artificial neural networks in its decision strategy, for capturing patterns embedded in data^52^, like the variational autoencoder^53^. In circumstances without prior knowledge or model for the channel and receiver imperfections, model-free reinforcement-learning techniques can be used to design quantum receivers^46,47^.

A few remarks on the optimization process of QREAL are worth making. A recent paper discovered that the initial parameters, to a large extent, determines the local minimum that a machine-learning algorithm converges to^54^. In our work, the QREAL formulator leverages a greedy algorithm to generate initial parameters that obey a preset criterion, locating near those of the CN receiver. Other sets of initial parameters may exist to further enhance the performance of QREAL, but as a tradeoff they would require more computing resources to identify. In addition, the initialization processes for QREALs tackling more complex quantum information processing problems would call for more systematic studies.

## Materials and methods

The classical computing part within the QREAL architecture is achieved by TensorFlow 2.1.0 package on Python 3.7 platform. The communication between classical computing and FPGA is held by TCP/IP protocol via wired local area network (LAN). The fiber-optical setup in front of a superconducting nanowire single photon detector (SNSPD) is compatible with the telecommunication c-band at 1550 nm right. The arrival time of the pulsed output from the SNSPD is recorded by the FPGA analog input port. The other FPGA analog input port is used for synchronization. Two analog output ports of the FPGA are properly amplified and connected to the amplitude and phase modulators. More details on the QREAL architecture and experimental setup are presented in Supplementary Information.

## Supplementary information

Supplementary Information for Quantum Receiver Enhanced by Adaptive Learning

## Data Availability

Supporting code and data has been uploaded to the online repository 10.5281/zenodo.7382847.
