# Supplementary material for: Quantum receiver enhanced by adaptive learning
Source: Light Sci Appl. 2022 Dec 8;11:344. doi: 10.1038/s41377-022-01039-5 (PMC9731947; doi:10.1038/s41377-022-01039-5)
Supplement: Supplementary file 1 — Supplementary Information for Quantum Receiver Enhanced by Adaptive Learning [file 41377_2022_1039_MOESM1_ESM.pdf]

Supplemental Information for Quantum Receiver Enhanced by Adaptive Learning

Chaohan Cui,[1] William Horrocks,[1] Shuhong Hao,[2] Saikat Guha,[1,3]
N. Peyghambarian,[1,2] Quntao Zhuang,[3,1,4] and Zheshen Zhang[2,3,1,5,*]

[1]*James C. Wyant College of Optical Sciences, University of Arizona, Tucson, AZ 85721, USA*
[2]*Department of Materials Science and Engineering, University of Arizona, Tucson, AZ 85721, USA*
[3]*Department of Electrical and Computer Engineering, University of Arizona, Tucson, AZ 85721, USA*
[4]*Department of Electrical and Computer Engineering, University of Southern California, Los Angeles, CA 90089, USA*
[5]*Department of Electrical Engineering and Computer Science, University of Michigan, Ann Arbor, MI 48109, USA*

I. DETAILED MODEL OF THE QREAL ARCHITECTURE

Hardware

In Fig. S1(a), The jth round entails a variational quantum circuit described by a reconfigurable unitary operation $\hat{U}_j(\alpha_j^{[k_1 k_2 \ldots k_{j-1}]})$, an ancillary quantum state $\hat{\rho}_{s_j}$, and a quantum measurement $\mathcal{M}_j$. To start the quantum-receiver operations, the first variational quantum circuit $\hat{U}_1(\alpha_1^0)$ ($[x_0] = 0$) takes the input in state $\hat{\rho}_{\text{in}}$ and the ancilla in state $\hat{\rho}_{s_1}$ and diverts a portion of its output quantum state to the measurement device. A control logic running on a classical processor then generates, in real time, a feed-forward signal $\alpha_2^{[k_1]}$ based on the measurement data k_1 so that the second variational quantum circuit is configured to $\hat{U}_2\left(\alpha_2^{[k_1]}\right)$. After N rounds of processing and measurements the classical processor selects an entry y from a decision table as the output of the quantum receiver. The control logic hence defines the quantum-receiver strategy.

Control logic

Figure S1(b) illustrates a control logic comprised of an M-ary decision tree with depth N and a decision table, both employed by a quantum receiver specified as QREAL(N, M). A node at the jth layer of the control logic carries a parameter $\alpha_j^{[x_{j-1}]}$ that specifies the setting of the variational quantum circuit in the jth round according to the measurement outcomes of previous $j-1$ rounds $[x_{j-1}]$. The control logic starts out at the root node ($j = 1$) and hops to a node in the next layer contingent upon the measurement outcome k_1, deriving the history records $[x_1] = [k_1]$. The migration in the decision tree continues until landing in an entry $[x_N] = [k_1 \ldots k_N]$ with $y([x_N]) \in \mathcal{D}$ in the decision table as the output of QREAL, where $\mathcal{D}$ is a set of

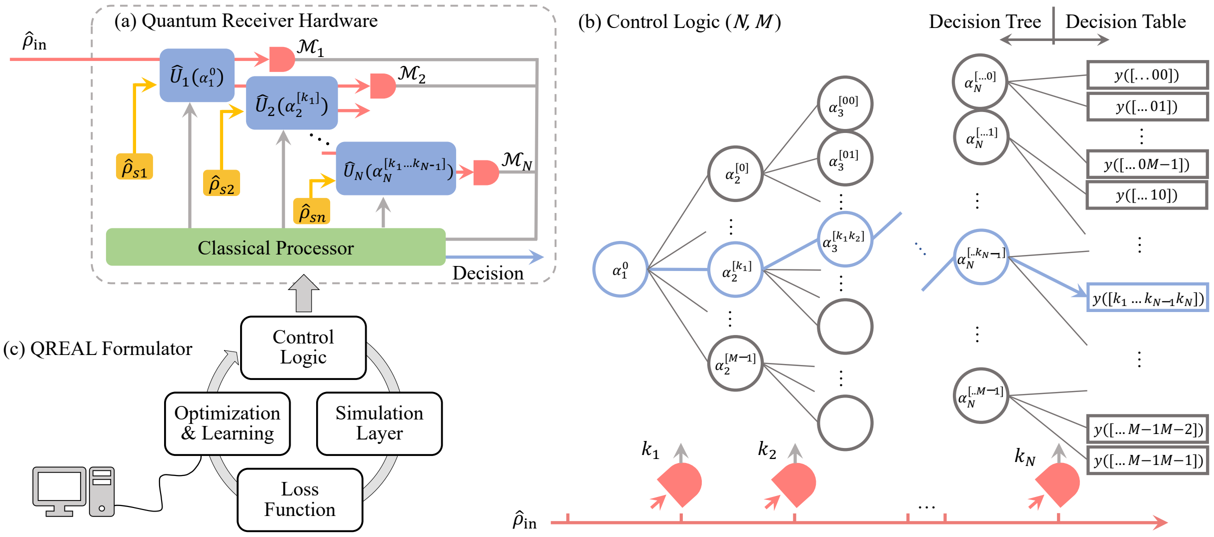

FIG. S1. A copy of Fig. 1 from the main paper: Overview on the QREAL architecture. (a) The hardware of a general quantum receiver composed of N rounds of processing. The jth processing round entails a variational quantum circuit represented by the unitary operation $\{\hat{U}_j\}$ configured by the parameter set $\{\alpha_j\}$, ancillary states ρ_{s_j}, and measurement apparatus $\mathcal{M}_j$. (b) The control logic comprised of a decision tree and a decision table. Blue lines show one possible path toward the output. Quantum-receiver output $\{y\}$ is determined by the sequence of measurement outcomes, tied to a unique path in the decision tree. (c) The QREAL formulator consisting of four modules (clockwise) as one iteration in the learning process. The control logic optimized by the QREAL formulator is compiled and built into the classical processor.

possible output values. An exemplar control-logic path is highlighted by the blue trajectory in Fig. S1(b).

Formulator

The QREAL formulator drawn in Fig. S1(c) aims to optimize the control logic for a specified QIP problem. The formulator iterates between a simulation layer, a loss function, and the optimization and learning algorithm to update the control logic. The simulation layer processes on the input density matrix and generates the measurement statistics characterized by the posterior probability distributions sampled during the execution of the quantum receiver. Deviations caused by noise and imperfections are also sampled through the simulation. The loss function is then derived from the measurement statistics to quantify the goal of optimization, such as the error rate. The classical optimization and learning algorithm subsequently updates the control logic based on the loss-function value and the gradient of its variables.

II. THEORY OF THE QREAL FOR WEAK COHERENT-STATE DISCRIMINATION

The objective of weak coherent-state discrimination is to minimize the error probability in labeling a quantum state belonging to the codeword set $\{|\beta_j\rangle\langle\beta_j|\}_{j=1}^{L}$ of L coherent states. A general description for the coherent-state ensemble is the density matrix $\hat{\rho}_{\rm in} = \sum_{i=1}^{L} P_i^{\rm in}|\beta_i\rangle\langle\beta_i|$, where $P_i^{\rm in}$'s are the prior probabilities. In the main text, the density matrix for the BPSK encoding with equal prior probabilities is $\hat{\rho}_{\rm in} = \frac{1}{2}|\beta\rangle\langle\beta| + \frac{1}{2}|-\beta\rangle\langle-\beta|$, whereas the density matrix for the QAM-6 encoding with equal prior probabilities is $\hat{\rho}_{\rm in} = \sum_{i=1}^{6}\frac{1}{6}|\beta_i\rangle\langle\beta_i|,\ \beta_i \in \{\beta e^{i\tan^{-1}1/2}, \beta e^{-i\tan^{-1}1/2}, i\beta/\sqrt{5}, -i\beta/\sqrt{5}, -\beta e^{i\tan^{-1}1/2}, -\beta e^{-i\tan^{-1}1/2}\}$. The mean photon number of BPSK is $|\beta|^2$ and of QAM-6 is $\frac{11}{15}|\beta|^2$ in front of the receiver. Each encoding picks a pure state $|\beta_i\rangle\langle\beta_i|$ from the codeword state set with the label $y_i^{\rm in} \in \boldsymbol{y}^{\rm in}$. In general, a noisy channel may contaminate the pure state, resulting a mixed state ρ_i at the quantum receiver.

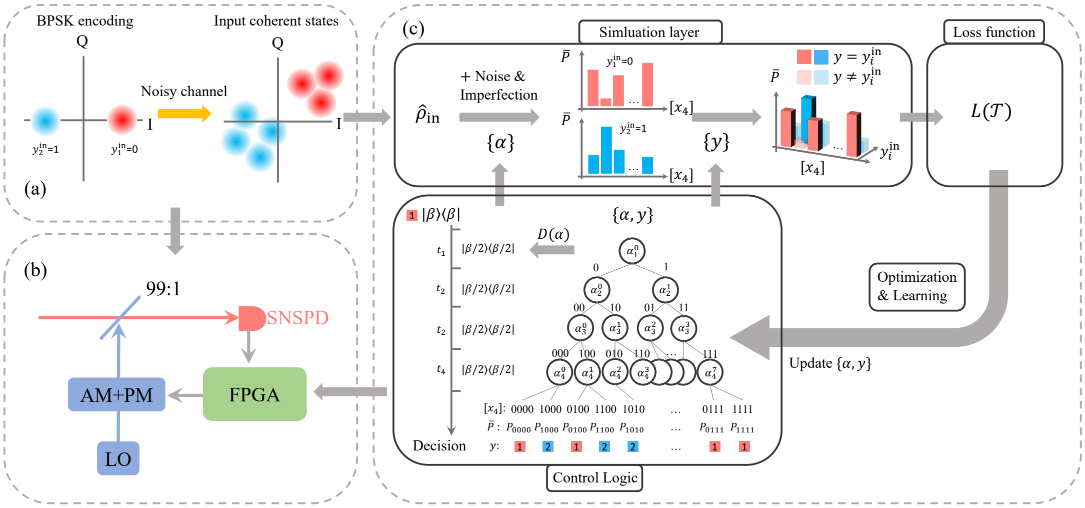

FIG. S2. QREAL(4,2) for BPSK. (a) Quantum-state evolution through a collective noisy channel. (b) The quantum-receiver for weak coherent-state discrimination. See Fig.S3(a) for more details. (c) The formulator for QREAL(4,2). $[x_4]$ is represented in decimal numbers. $\{\alpha_j^{[x_j]}\}$ is the parameter set for displacements at the jth round. $\{[x_4]\}$ is the history of measurement outcomes. $\{\bar{P}\}$ is the probability distribution associated to input state $|\beta\rangle\langle\beta|$ and the history $[x_4]$. $\{y\}$ is the decision based on $\{[x_4]\}$.

The variational quantum circuit for the jth round of the quantum-receiver hardware sketched in Fig. S2(b) consists of a beamsplitter tapping $1/N$ portion of the input quantum state $\hat{\rho}_{\text{in}} = |\beta\rangle\langle\beta|$ followed by a reconfigurable quadrature displacement operation $\hat{U}_j(\alpha_j) = \exp(\alpha_j \hat{a}^\dagger - \alpha_j^* \hat{a})$ on the tapped coherent state $|\beta/\sqrt{N}\rangle$. A photon-number resolving (PNR) detector then measures the displaced state $|\beta/\sqrt{N} + \alpha_j\rangle$, yielding k_j counted photons as its output. The classical processor subsequently collects the output from time-multiplexed PNR detector and sets the amount of displacement applied in the variational quantum circuit of the next round.

Suppose that the duration of the input quantum state is T seconds, the depth of control logic is N, and the PNR can resolve up to $M-1$ photons. The jth round is executed over t_j seconds so that $T = \sum_{j=1}^{N} t_j$. The measurement outcome $k_j \in \{0, 1, ..., M-2, M-1\}$ determines the route to the next node in the decision tree. The measurement outcomes in the first j rounds is assembled to form the sequence $[x_j] \equiv [k_1 k_2 ... k_{j-1} k_j],\ j \leq N$.

The effect of noise and other imperfections is modeled by a probability deviation f_N, which in general is a placeholder that can accommodate any noise patterns and can be learned from interaction with the quantum hardware. In our experiment, f_N depends on the input coherent state $|\beta_i\rangle\langle\beta_i|$, the displacement $\alpha_j^{[x]}$ applied in the jth round, the duration of the jth round t_j, and the measurement outcome k_j. To account for the randomness of the noise and the stochastic feature of the system, f_N is sampled stochastically and added to a batch of decision trees without resorting to a full analytical model. The model shown here is for a general description while the details of our implementation is discussed in the next section. With the input coherent state $|\beta_i\rangle\langle\beta_i|$ labeled by y_i^{in}, the conditional probability of attaining k_j in the PNR measurement reads

$$P_j(k_j \mid y_i^{\text{in}}, \alpha_j^{[x_{j-1}]}, f_N) = \begin{cases} e^{-\gamma_{ij}} \gamma_{ij}^{k_j}/k_j! + f_N\left(\alpha_j^{[x_{j-1}]}, \beta_i, t_j, k_j\right), & k_j \in \{0, 1, ..., M-2\} \\ 1 - \sum_{k'=0}^{k'<M-1} P_j\left(k' \mid \alpha_j^{[x_{j-1}]}, y_i^{\text{in}}, f_N\right), & k_j = M-1 \end{cases} \tag{S1}$$

where

$$\gamma_{ij} = |\beta_i \sqrt{t_j/T} + \alpha_j^{[x_{j-1}]}|^2 \tag{S2}$$

The size-D batch-averaged probability of recording a specific sequence of $[x_N]$ conditioned on the input-state label y_i^{in}, sampled noise function $f_N \in \mathcal{D}$, and the displacement settings of all N rounds, defined as $\boldsymbol{\alpha}_N^{[x_{N-1}]} \equiv \left\{\alpha_1^0, \alpha_2^{[x_1]}, \alpha_3^{[x_2]}, ..., \alpha_N^{[x_{N-1}]}\right\}$, along the decision path set by $[x_N]$ is thus

$$\bar{P}\left([x_N] \mid y_i^{\text{in}}, \boldsymbol{\alpha}_N^{[x_{N-1}]}\right) = \frac{1}{D} \sum_{f_N \in \mathcal{D}} P_1(k_1 \mid \alpha_1^0, y_i^{\text{in}}, f_N) \prod_{j=2}^{N} P_j\left(k_j \mid y_i^{\text{in}}, \alpha_j^{[x_{j-1}]}, f_N\right) \tag{S3}$$

The output of QREAL conditioned on the sequence of measurement outcomes is given by the label of the input coherent state that maximizes the conditional probability of registering this sequence:

$$y\left([x_N] \mid \boldsymbol{\alpha}_N^{[x_{N-1}]}\right) = \arg\max_{y_i^{\text{in}}} P_i^{\text{in}} \bar{P}\left([x_N] \mid y_i^{\text{in}}, \boldsymbol{\alpha}_N^{[x_{N-1}]}\right) \tag{S4}$$

The loss function L in turn is defined as the overall error rate given the entire decision tree $\mathcal{T}$, defined as $\mathcal{T} \equiv \left\{\boldsymbol{\alpha}_N^{[x_N - 1]}\right\}$, over all $[x_N]$.

$$L(\mathcal{T}) = \sum_{[x_N], i} \left(1 - \delta_{y_i^{\text{in}}, y\left([x_N] \mid \alpha_N^{[x_{N-1}]}\right)}\right) P_i^{\text{in}} \bar{P}\left([x_N] \mid y_i^{\text{in}}, \boldsymbol{\alpha}_N^{[x_{N-1}]}\right) \tag{S5}$$

where P_i^{in} is the prior probabilities for each input quantum state, and the Kronecker delta function $\delta_{y_i^{\text{in}},y}$ ensures that only the possibilities for incorrect decisions that fail to identify the input quantum-state label contributes to the loss function.

The above algebras fulfill the requirement of automatic differentiation needed by backpropogation, which is exploited in the chosen machine-learning algorithm to optimize QREAL's control logic by gradient until landing in a local minimum of the loss function.

The machine-learning algorithm leverages stochastic gradient descent with adaptive momentum. The initial control logic and decision table are both picked by a greedy strategy. This greedy strategy employs stochastic optimization over the displacement phases while fixing the magnitude so that it tends to nullify the most-likely quantum state. The initial control logic and decision table are identical to these of the CN receiver in the absence of phase noise, but start to deviate from these of the CN receiver when phase noise becomes prominent.

A complete illustration for the QREAL(4,2) formulator for BPSK with equal t_j's is drawn in Fig. S2(c).

The relative entropy between measurement statistics associated with two incoming quantum-state labels y_p^{in} and y_q^{in} upon the completion of the n round is estimated by

$$D_{\text{KL}}(p||q;n) = \sum_{[x_n]} \bar{P}\left([x_n] \mid y_p^{\text{in}}, \boldsymbol{\alpha}_n^{[x_{n-1}]}\right) \log_2 \left(\frac{\bar{P}\left([x_n] \mid y_p^{\text{in}}, \boldsymbol{\alpha}_n^{[x_{n-1}]}\right)}{\bar{P}\left([x_n] \mid y_q^{\text{in}}, \boldsymbol{\alpha}_n^{[x_{n-1}]}\right)} \right) \tag{S6}$$

A larger relative entropy renders two quantum states more distinguishable, thereby leading to less ambiguity and lower error rates.

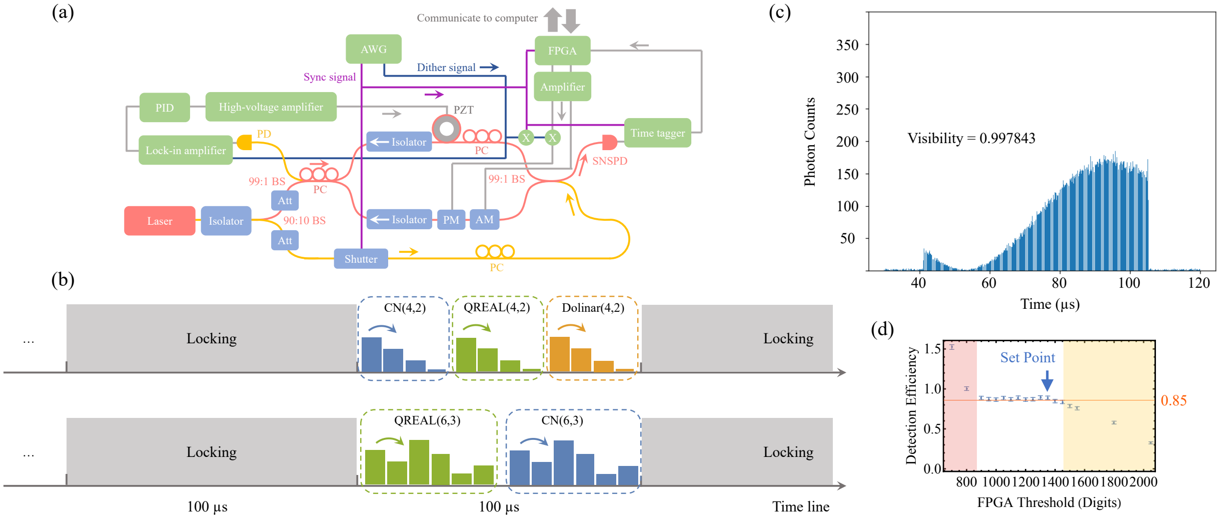

FIG. S3. (a) Experimental setup. The quantum-receiver hardware platform includes a Mach-Zehnder interferometer (red), a phase-locking module (yellow), fiber-optic components (light blue), electronic devices (green), electrical synchronization signals (magenta), the dither (dark blue), and control signals (gray). (b) The timelines of phase-locking and data-aquisition stages for (4,2) receivers and (6,3) receivers. All quantum-receiver schemes are tested in a time-multiplexed sequence subject to nearly the same noise condition. (c) The histogram of interference visibility recorded in 5s of a 100 μs span by linearly scanning PM in the phase-locking state. The histogram is a screenshot of the hardware monitor. Similar statistical charts are used for characterising noise. (d) Detector efficiency vs FPGA threshold. Threshold set point is chosen after calibration.

III. EXPERIMENTAL SETUP AND CALIBRATION

The experiment entails the QREAL formulator and the quantum-receiver hardware that routinely communicate via an ethernet cable. After modeling the noise patterns through estimation on the hardware, the QREAL formulator running on a desktop computer continuously learn and deliver the optimized control logic to the classical processor of the quantum-receiver hardware. The classical processor consisting of an FPGA operates the control logic and collects the data produced by the quantum-receiver hardware.

Noise estimation

We first discuss the model for the noise. In the current experiment, the chosen model for f_N contains three stationary stochastic processes. Eq. (1) then becomes

$$P_j(k_j \mid y_i^{\text{in}}, \alpha_j^{[x_{j-1}]}) = \begin{cases} e^{-\tilde{\gamma}_{ij}} \tilde{\gamma}_{ij}^{k_j} / k_j!, & k_j \in \{0, 1, ..., M-2\} \\ 1 - \sum_{k'=0}^{k'<M-1} P_j \left(k' \mid \alpha_j^{[x_{j-1}]}, y_i^{\text{in}}, f_N \right), & k_j = M-1 \end{cases} \tag{S7}$$

(S8)

where

$$\tilde{\gamma}_{ij} = |\beta_i \sqrt{t_j/T} + (1+\Delta) e^{i\delta} \alpha_j^{[x_{j-1}]}|^2 + P_{\text{dc}} * t_j \tag{S9}$$

Phase-locking noise δ and amplitude-modulation percentage errors Δ are treated as two sampled collective noise as Gaussian variables added to each round of decoding. The last constant Poissonian parameter P_{dc} accounts for the degradation by dark counts and ambient light.

To initialize the QREAL formulator, the FPGA runs an estimation procedure to infer the average number of received photons, fringe visibility, and noise characteristics of the hardware. The estimation procedure probes the system by measuring millions of displacements on a single code. Then, the statistics of the output are sent to the QREAL formulator to estimate the variances of the two Gaussian distributions and the mean of the Poissonian distribution. In detail, P_{dc} is estimated by setting the signal and LO to vacuum. Upon knowing P_{dc}, the phase and amplitude variances are fit to the photon-counting statistics as the displacement is swept, from nullifying the signal to amplifying the signal. Then, the QREAL formulator can sample Δ and δ independently for each decision tree and optimize the strategy collectively by averaging a batch of decision trees.

The QREAL formulator

The QREAL formulator is programmed in Python using TensorFlow, an open-source machine-learning library that supports parallel computing, symbolic graph computing, and automatic differentiation. The employed batch sizes are 1000 for BPSK and 100 for QAM-6.

Prior to the iterative learning procedure, the QREAL formulator first uses a greedy algorithm to create an initial control logic composed of a decision tree that carries the displacements applied by the variational quantum circuits and a decision table that stores the outputs of the QREAL. During the iterations, the simulation layer simulates the evolution of batches of input quantum states through the variational quantum circuits configured by the current control logic with sampled noise and derives the measurement outcomes in all rounds using the coherent-state discrimination model (Section II), based on which the posterior probability distribution for each input quantum state from the codeword set is formed. Each entry in the decision table ties to a distinct sequence of measurement outcomes and labels the input quantum state that yields the

largest posterior probability given that sequence. Next, the loss function and its gradients to the control-logic parameters are calculated based on the simulated probability that QREAL's output differs from the input quantum-state label, i.e., the error rate. Finally, the optimization and learning module updates the control logic by adjusting its parameters toward a reduced error rate. The updated control logic will be used in the simulation layer in the next iteration until the loss function converges.

Hardware platform

The variation quantum circuit is a displacement operation implemented by an interference between the quantum-signal light and a phase-stabilized strong local oscillator (LO) on a 99:1 beamsplitter, as sketched in Fig. S2(b). The amplitude and phase modulators in the LO arm are controlled by a field-programmable gate array (FPGA), which processes the measurement data from a superconducting nanowire single-photon detector (SNSPD) to follow the QREAL control logic. Achieving a high overall efficiency of the circuit is critical to beat standard quantum limit (SQL). We remove all lossy components between the displacement operation and the detector, resulting in the highest efficiency ($\sim$85%) reported to date. Meanwhile, the system achieves high visibility ($>$99.7%) without trading its efficiency by exploiting back-propagation locking.

The quantum-receiver hardware platform illustrated in Fig. S3(a) operates at 1550 nm for compatibility with fiber-optic networks. Due to the required stable relative phase between the LO and and quantum-signal light, the system switches periodically between a phase-locking stage and the data-acquisition stage, each consuming 100 μs, as illustrated in (Fig. S3(b)). In the phase-locking stage, a shutter is opened to inject counter-clockwise locking light (orange) into the Mach-Zehnder interferometer. A dither loop stabilizes the phase difference between the LO and the quantum-signal light. The dither produced by a lock-in amplifier introduces phase modulation at 31.75 kHz. The same lock-in amplifier demodulates the output from the photo diode detecting the locking light to generate the error signal, which is subsequently processed by a PID and amplified by a high-voltage amplifier to drive a piezoelectric fiber stretcher that compensates for the phase difference. In the data-acquisition stage, the counter-clockwise locking light and the electronic dither are both blocked, the quantum-signal light is launched, controlled by the FPGA, detected by an SNSPD followed by a time tagger. A 5 kHz signal synchronizes the FPGA, the shutter, the modulators, and the time tagger.

The phase-locking fluctuation, the ambient light, the initial calibration errors, and the SNSPD dark counts introduces noise to the system. We calibrate the mean photon number, noise, and other imperfections by tracking the photon histograms while scanning the LO phase and amplitude, which in turn inform the initialization and noise samples for the QREAL formulator. The construction of the histogram is triggered by the synchronization signal. Each histogram is accumulated over 5 seconds. Our setup maintains a visibility over 99.75%, shown in a screenshot of the histogram monitor in Fig. S3(c).

The detection efficiency is dependent on the FPGA threshold set to read the electronic pulses from SNSPD. The connection between the FPGA threshold and detector efficiency is exhibited in Fig. S3(d). Low thresholds in the red shade result in overcounting the SPSPD pulses. In contrast, high thresholds in the orange shade undercounter the SNSPD pulses, thereby reducing the overall efficiency. By appropriately choosing the FPGA threshold, the overall efficiency is calibrated to be $\sim$ 85%. The PNR detection is realized by time-multiplexed on-off detection in a 5 μs windows, which is much longer than the 25 ns detector deadtime.

In the experiment, the BPSK format is tested for a continuous duration of 800 seconds, recording 4 million samples of each design at a given mean photon number. QAM-6 is tested for a continuous duration of 1200 seconds, obtaining 6 million samples for each QREAL design at a given mean photon number. All the data are first recorded in the FPGA and then analyzed offline.

IV. FPGA CONFIGURATIONS AND CHARACTERISTICS

We choose the STEMlab 125-14 RedPitaya board to develop the QREAL. To keep the latency at a minimum, all necessary data (ML optimized voltages and count values) are stored in the programmable logic

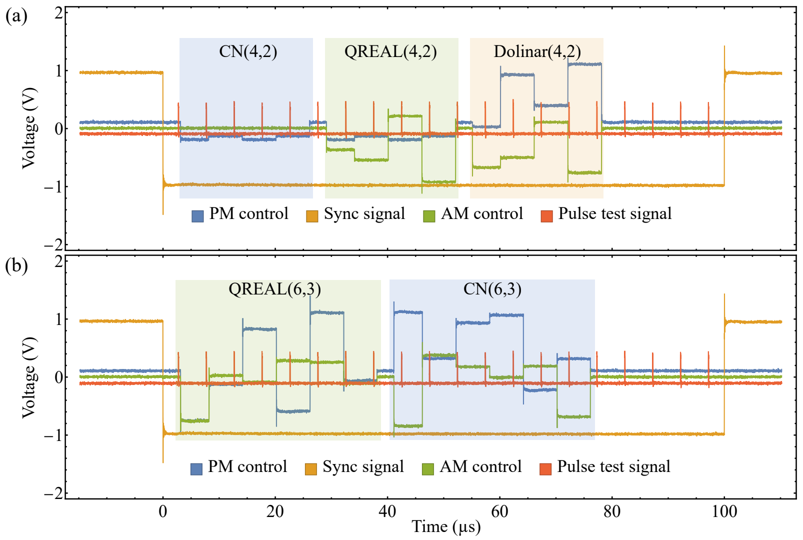

FIG. S4. (a) A sample of receiver(4,2) FPGA runtime patterns during logic tests. (b) A sample of receiver(6,3) FPGA runtime patterns during logic tests. PM control voltage and pulse test signal are shifted up and down, respectively, for better visualization. By setting the inter-bin buffer to around 1 μs (125 clock cycles), ringing artifacts are avoided, and the search through LUT is guaranteed. There is also an inter-frame buffer between different receiver designs for switching LUTs and recording results. The pulse test signal is produced by the second FPGA and triggered by the sync signal. The test pulse repetition rate is a runtime variable for traversing all possible routines of quantum receivers.

(PL) block RAM. Approximately 7% of the available 36 kbit block RAM tiles (4 of the total 60) are used by the lookup table (LUT) entries and count data. Given the low block RAM usage, more memory-intensive applications can be readily implemented using our PL design. Interactions with the block RAM are achieved using C programs, custom memory reading/writing cores, and combinations of basic tasks, such as updating the LUT, recording data for a user-entered amount of time, and writing the count data to a designated file, are automated with shell scripts. Parameters such as bin size, inter-bin buffers, inter-frame buffer, detection threshold, recording time are runtime configurable via GPIOs. All custom bitstreams are developed in Verilog by Vivado v2020.1 using open-source projects as templates and augmented using custom cores to achieve our desired performance.

Components of the QREAL design are verified in Vivado's simulation environment, in field tests, or a combination of both. The SNSPD output is characterized using the RedPitaya board, and multiple schemes are tested to guarantee accurate pulse detection. After implementing and testing multiple detection algorithms in the field, the threshold-based decision logic is chosen. A pulse is detected if the input voltage is below the user entered threshold on one clock cycle and above it the next. The threshold value is experimentally chosen and tuned for our setup. With an attenuated laser connected to the SNSPD, the average photon count per second is seen to be in reasonable agreement with the commercial time tagger measurement. After characterizing the detection response of the SNSPD, we use a separate FPGA to simulate the trigger signal and a custom SNSPD response. The period and amplitude of both outputs, along with pulse width, duration, and detection pattern, are configurable at runtime, shown in Fig. S4. The above feature enables us to verify the logic flow of each type of receiver before implementing it to the quantum-receiver hardware platform.

V. QREAL'S PERFORMANCE VS. PREVIOUS SETUPS FOR BPSK

The QREAL's performance is benchmarked with previous demonstrations of decoding BPSK in the following table.

	Efficiency	MPN range to beat SQL	ER @ MPN=1†	Detection	λ
Cook, Martin, & Geremia [1]	35%	<0.25	12%	On/off	852 nm
DiMario & Becerra [2]	72%	< 0.5	3%	PNR	633 nm
QREAL(4,2) (This work)	85%	< 1.6 *	1.6%	On/off *	1550 nm

TABLE I. Comparison between state-of-the-art experimental results for quantum receivers for BPSK encoding. The "ER @ MPN = 1" column shows the lowest experimental error rates at mean photon number 1. Fringe visibility of more than 99.7% achieved in all platforms. MPN: mean-photon-number; ER: error rate. (†) Experimental error rate at a mean photon number around one. (*) The introduction of PNR detectors can extend the mean-photon-number range by several folds according to simulations in Ref. [2].

VI. ADDITIONAL INFORMATION

This supplementary information accompanies the manuscript on the Light: Science & Applications website `https://www.nature.com/lsa/`.

* zszh@umich.edu

[1] Robert L. Cook, Paul J. Martin, and John M. Geremia. Optical coherent state discrimination using a closed-loop quantum measurement. *Nature* **446**(7137), 774–777, 2007.

[2] M. T. DiMario and F. E. Becerra. Robust measurement for the discrimination of binary coherent states. *Phys. Rev. Lett.* **121**(2), 023603, 2018.
